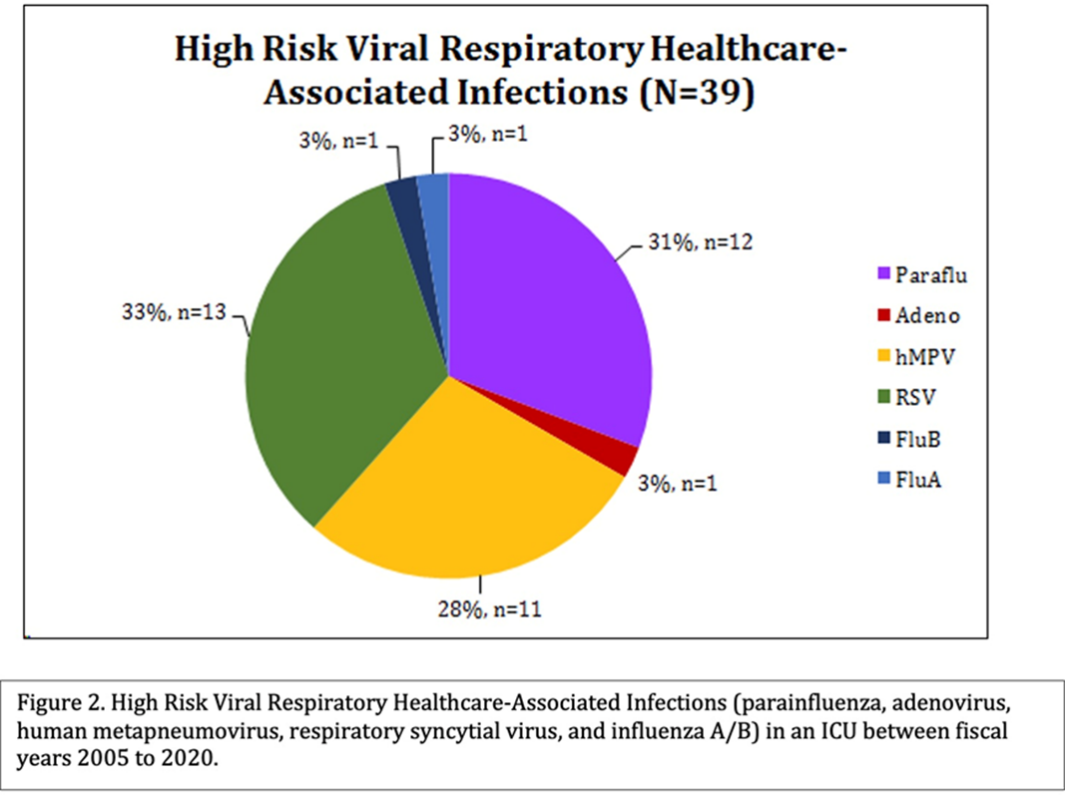# Healthcare-Associated Viral Respiratory Infections in a Pediatric Intensive Care Unit and Cardiovascular Intensive Care Unit

**DOI:** 10.1017/ash.2021.148

**Published:** 2021-07-29

**Authors:** Kelly Feldman, Jasjit Singh, Wendi Gornick

## Abstract

**Background:** Healthcare-associated infections (HAIs) affect patient health and are tracked closely by infection prevention. Patients in a pediatric intensive care unit (PICU) acquired viral respiratory infections had longer use of respiratory support. We sought to determine the types of viral respiratory HAIs (VR-HAIs) acquired in the PICU and the characteristics of those affected. **Methods:** CHOC Children’s Hospital is a 334-bed tertiary-care center. Charts were reviewed on patients with VR-HAIs from fiscal years (FY) 2005–2020. High-risk VR-HAI (HR-VR-HAI) were influenza A and B, respiratory syncytial virus (RSV), adenovirus, parainfluenza, and human metapneumovirus (hMPV, added in FY 2014). Patients in the PICU, cardiovascular ICU (CVICU), and oncology ICU (OICU) with HR-VR-HAIs were reviewed. Patients were categorized according to underlying pathology, immunosuppression, and isolation prior to HR-VR-HAI. Increased respiratory support was defined as any increase from a patient’s baseline support ±24 hours of viral diagnosis: increase in oxygen flow or transition from nasal cannula to high-flow nasal cannula or ventilator support. Antibiotic escalation, defined as initiation of antibiotic therapy for ≥2 days ±24 hours of viral diagnosis or broadening the spectrum of antimicrobials for ≥2 days ±24 hours of viral diagnosis. **Results:** During FY 2005–2020, there were 204 VR-HAIs: 143 HR-VR-HAIs (70%), of which 39 (27.2%) occurred in ICUs (Figure 1). Most of the HR-VR-HAIs were RSV, parainfluenza, and hMPV (Figure 2). Of 39 patients, 10 (25.6%) had underlying oncologic conditions, 9 of whom were immunosuppressed. Of 39 patients, 16 (41%) had structural cardiac disease, 4 (10.3%) had pulmonary disease, 5 (12.8%) had neurologic disease, and the remaining 4 (10.3%) had other comorbidities. Of 39 patients, 12 (31%) required an increase in respiratory support and 13 (33%) had escalation of antibiotics. Of 39 HR-VR-HAI patients, 2 died within 2 weeks of acquisition. **Conclusions:** HR-VR-HAIs are uncommon in ICUs. RSV, parainfluenza, and hMPV are the most common, and 1 of 3 of patients required escalation in respiratory support and/or escalation in antibiotics. All patients had underlying comorbidities. In our series, there were 2 deaths within 2 weeks of infection.

**Funding:** No

**Disclosures:** None

**Figure 1.**
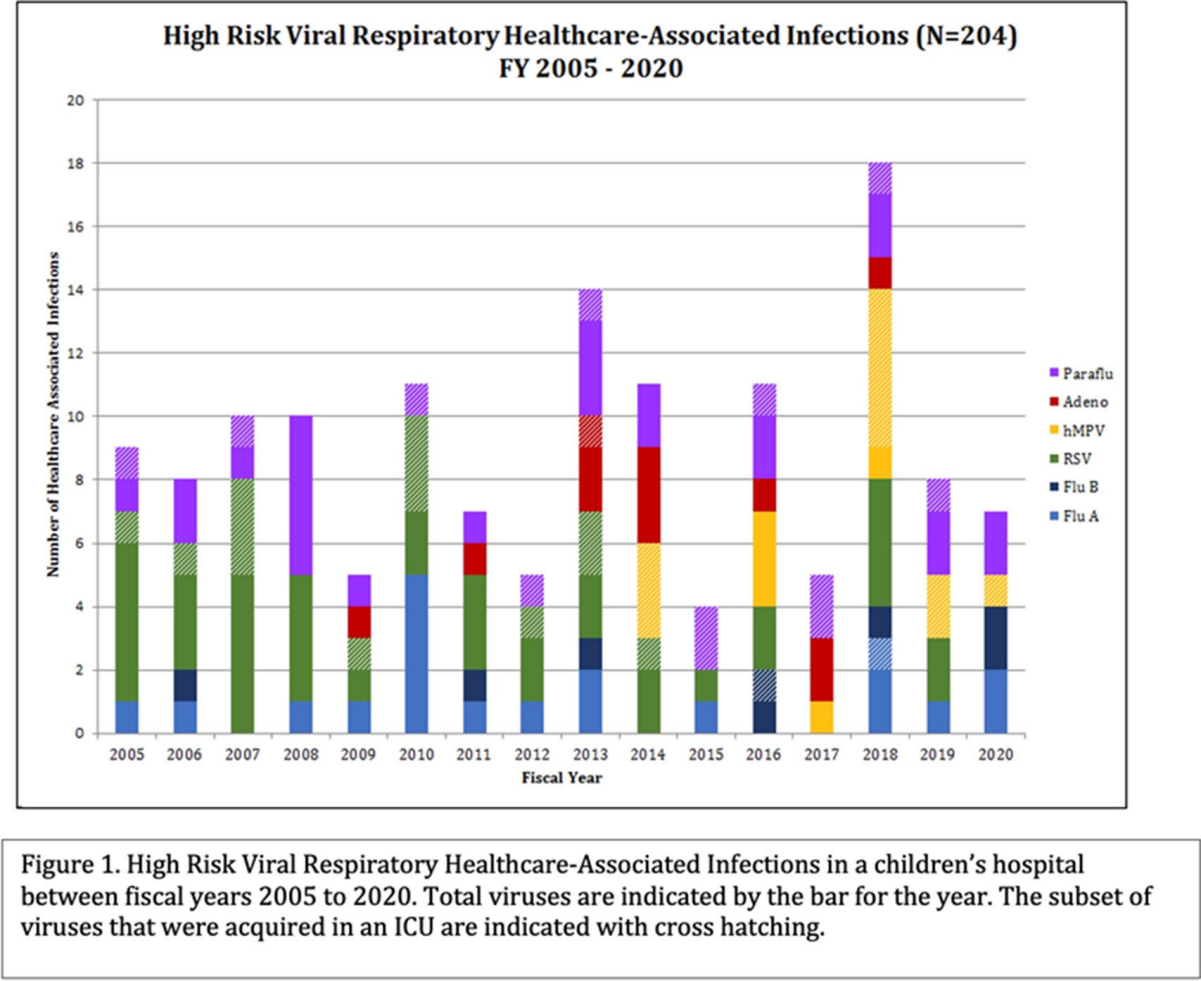

**Figure 2.**